# The Hypothalamus-Pituitary-Adrenal Axis and Social Cognition in Borderline Personality Disorder

**DOI:** 10.2174/1570159X21666230804085639

**Published:** 2023-08-07

**Authors:** Eugenia Kulakova, Livia Graumann, Katja Wingenfeld

**Affiliations:** 1 Charité - Universitätsmedizin Berlin, Corporate Member of Freie Universität Berlin and Humboldt-Universität zu Berlin, Klinik für Psychiatrie und Psychotherapie, Campus Benjamin Franklin, Berlin, Germany

**Keywords:** Borderline personality disorder, stress, hypothalamus-pituitary-adrenal axis, cognition, social cognition, major depressive disorder

## Abstract

Borderline personality disorder (BPD) is characterized by emotional instability, impulsivity and unstable interpersonal relationships. Patients experience discomforting levels of distress, inducing symptoms like dissociation, aggression or withdrawal. Social situations are particularly challenging, and acute social stress can reduce patients’ cognitive and social functioning. In patients with Major Depressive Disorder or Posttraumatic Stress Disorder, which show high comorbidity with BPD, the endocrine stress response is characterized by Hypothalamus-Pituitary-Adrenal (HPA) axis dysfunction, which affects cognitive functioning. Compared to these clinical groups, research on HPA-axis function in BPD is relatively scarce, but evidence points towards a blunted cortisol reactivity to acute stress. Since BPD patients are particularly prone to social stress and experience high subjective difficulties in these situations, it seems plausible that HPA-axis dysregulation might contribute to decreased social cognition in BPD. The present review summarizes findings on the HPA-axis function in BPD and its association with social cognition following acute social stress. For this purpose, we review literature that employed a widely used social stressor (Trier Social Stress Test, TSST) to study the effects of acute social stress on social cognition and the HPA-axis response. We contrast these findings with studies on social cognition that employed Cyberball, another widely used social stressor that lacks HPA-axis involvement. We conclude that research on social cognition in BPD reveals heterogeneous results with no clear relationship between social functioning and HPA-axis response. More research is needed to better understand the psychophysiological underpinnings of impaired social cognition in BPD.

## 1 BORDERLINE PERSONALITY DISORDER: CLINICAL FEATURES

Borderline personality disorder (BPD) is a severe mental illness with a high burden of disease. Among psychiatric populations, it is the most frequent personality disorder, with a prevalence of around 22% in inpatient and 10% in outpatient settings [1]. Patients with BPD often suffer from comorbid mental disorders, most prominently Major Depressive Disorder (MDD) and Posttraumatic Stress Disorder (PTSD) [2]. In addition, BPD is associated with a range of medical illnesses, including cardiovascular diseases and obesity [3].

Besides emotional instability and impulsivity, BPD is characterized by a pervasive pattern of instability in interpersonal relationships, fear of abandonment and chronic feelings of emptiness [4]. Patients experience intense and rapidly changing mood states and high levels of distress, which can lead to aggressive, self-injurious or suicidal behaviors and transient dissociative symptoms. While fear of abandonment and disturbed relationships seem to remain stable over time [5], many of the symptoms, such as anger, impulsivity and suicidal attempts, mainly occur and worsen under acute stress [6, 7]. Etiological models link BPD to early life stress and adversity, such as emotional invalidation [8] and childhood abuse or neglect [9]. Symptoms typically emerge in adolescence or young adulthood and commonly remit by early middle age. However, long-lasting interpersonal problems such as poor social integration remain after general symptom remission [10]. Several psychotherapeutic approaches, *e.g*., Dialectical Behavior Therapy (DBT) and Mentalization-based Therapy (MBT) [11-14], target such social difficulties. Besides stress tolerance and emotion regulation, training of interpersonal skills is one of the main components of DBT.

Acute stress plays an important role in the ongoing perpetuation and exacerbation of BPD symptoms [5]. Several studies suggest that BPD patients experience more stress in their daily lives than healthy controls [15, 16]. The acute stress response of BPD patients can be characterized by either fight-or-flight behavior, such as interpersonal aggression or withdrawal (often occurring in quick succession) or freezing, *i.e*., transient dissociations. These response patterns are associated with a drastically shortened window of adaptive behavioral control, which might hint at temporarily reduced levels of cognitive control and social cognition. Especially in social interactions, this reactivity further promotes interpersonal problems as part of the symptomatology, resulting in a vicious circle that leads to poor social integration.

As such, there is ample evidence that acute stress plays an important role in the difficulties BPD patients experience in social situations. In the present article, we will review the existing literature on the influence of acute psychosocial stress on social cognition in patients with BPD. First, Section 2 will give a general overview of the physiological stress systems, in particular the Hypothalamus-Pituitary-Adrenal (HPA)-axis and its influence on cognition in healthy and clinical populations. Section 3 will summarize the impact of stress on social cognition and review the effects of acute social stress on social cognition in BPD. To review the potential influence of the HPA axis on social cognition in BPD, we will focus on two prominently used stressors in clinical research - the Trier Social Stress Task (TSST) and the Cyberball task. Section 4 will provide a short summary and conclusion.

## 2 THE HYPOTHALAMUS-PITUITARY-ADRENAL (HPA) AXIS AND COGNITION

Acute stress is a transient response to a challenging or threatening situation (the stressor) with the goal of overcoming the challenge and restoring homeostatic balance. Appraising a certain situation as stressful activates several physiological and psychological processes. The two most prominent biological systems responsible for the stress response are the fast-acting vegetative autonomic nervous system (ANS) and the slow-acting neuro-endocrinal hypothalamic-pituitary-adrenal (HPA) axis. Even though the stress response is constituted by a complex interaction between these and more systems (including endocannabinoid and oxytocin signaling), this review focuses on the stress-induced activation of the HPA-axis, as it is known to consistently affect cognitive processes as well as social cognition.

Importantly, stress, including adverse experiences in childhood and adulthood, dramatically increases the risk of developing mental and somatic disorders. Alterations of the HPA axis have been widely studied in several mental disorders. It, therefore, seems plausible to assume that the HPA axis also plays a role in the acute symptomatology of BPD.

This section will (2.1) give an introduction to HPA axis functioning, (2.2) describe its effects on cognition, and then (2.3) summarize known HPA axis alternations in MDD and PTSD, the most prominent comorbidities of BPD, as well as (2.4.) in BPD.

### 2.1 The HPA-axis Stress Response

The stress response is initiated by the appraisal of an external or internal state as challenging and threatening. This allows a certain leeway regarding the interpretation of stimuli that can show inter-individual differences or symptomatic biases associated with mental illness. However, once initiated, the HPA-axis-mediated stress response proceeds in a clear sequence. Briefly, upon stress exposure, corticotropin-releasing factor (CRF) is released from the hypothalamus and transported to the anterior pituitary, where it stimulates the secretion of adrenocorticotropin (ACTH) into the bloodstream. ACTH, in turn, stimulates the synthesis and release of glucocorticoids (GCs) from the adrenal cortex. The HPA-axis is counter-regulated *via* negative feedback mechanisms by circulating GCs targeting the pituitary and the hypothalamus, but also brain regions such as the hippocampus and the prefrontal cortex (PFC). The hippocampus is rich in GC receptors and exerts negative feedback on the paraventricular nucleus of the hypothalamus, thereby reducing the activity of the HPA axis. This negative feedback loop is essential for the efficient regulation of the HPA axis [17].

Within the central nervous system, GCs bind to two subtypes of intracellular receptors, the mineralocorticoid receptor (MR) and the glucocorticoid receptor (GR). They differ in their affinity and distribution within the brain: while GR is expressed throughout the whole brain, MR is mainly located in limbic brain areas, *e.g*., the hippocampus [17]. Given their expression in relevant brain regions, it is not surprising that GC has been shown to play a role in the regulation of cognitive and emotional processes.

### 2.2 The HPA-axis and Cognition

Stress and cognition show an inverted U-shaped relation: mild stress can improve cognitive functions such as attention, executive control, memory and reasoning, while strong or chronic stress leads to reduced cognitive functions that rely on frontal and hippocampal processing, *i.e*., regions showing the strongest GC receptor density [18]. It has been suggested that stress leads to a flip from reflective to reflexive control of behavior due to the weakened function of the PFC [19]. Contrary, tasks that rely on implicit memory of well-trained, automatic functions realised in the amygdala or striatum might keep functionality during high or chronic stress [20].

Many of the known cognitive effects of mild and transient stress can be attributed to HPA-axis functioning. Due to their prominence throughout the brain, corticoid receptors modulate cognitive processes such as attention, concentration, learning and memory [21, 22]. Possibly the most consistent findings are the enhancing effects of GCs on learning and memory consolidation while at the same time impairing memory retrieval [22, 23]. Most of these GC effects on cognition have been attributed to GRs, which likely regulate the normalization of stress-induced effects and storage of (stress-related) information for future use [24, 25]. However, MR signaling also has important effects on cognition [26-28]. There is some evidence from human and animal studies that brain MRs affect attentional vigilance to salient information, the appraisal of novel situations, behavioral flexibility and decision-making. MR blockade impairs memory and executive function in healthy humans [29-31]. Inversely, after stimulating the MR with its agonist fludrocortisone, memory encoding, working memory and visuospatial memory improve [32-34]. Thus, the MR is particularly important for the early phase of the stress response as well as rapid cortisol effects on appraisal and cognitive processes [35].

Taken together, MRs and GRs are expressed in different brain regions and play different roles during the acute stress response. While MRs, in concert with the noradrenergic stress response, shift information processing to salient information, GRs reduce the initial stress response to prevent its overshoot and restore homeostasis [26, 36, 37].

### 2.3 The HPA-axis and Cognition in MDD and PTSD

Several mental disorders are characterized by alterations of the HPA axis. In this section, we will focus on Major Depressive Disorder (MDD) and Posttraumatic Stress Disorder (PTSD) because both conditions show a high prevalence of BPD [2]. MDD has been characterized by an increased cortisol release, a reduced feedback sensitivity of the HPA-axis and decreased GR sensitivity [38-41]. Interestingly, several studies have found impaired cognition to be associated with elevated cortisol in patients with MDD - a pattern that contrasts with the cognition-improving effects observed in healthy participants [42-46]. However, not all studies concur [47, 48].

In a series of studies, our group investigated how a single administration of 10 mg hydrocortisone affected several neuropsychological domains in MDD. In a declarative memory task, cortisol impaired memory retrieval in healthy participants but not in MDD patients [49]. A similar pattern was observed for autobiographic [50] and working memory [51]. With respect to response inhibition, a measure of executive function, cortisol improved performance in healthy individuals but again did not affect MDD patients [52]. These results indicate that hippocampus and PFC-based cognitive processes were not affected by cortisol in MDD patients, which is in line with reduced GR sensitivity in MDD.

While fewer studies have focused on MR alterations in MDD, there is some evidence of decreased MR expression in the hippocampus and PFC [53, 54]. A study with relatively young participants found equally improved verbal memory and executive function after MR stimulation in both MDD and healthy controls [55]. However, older MDD patients performed worse in verbal learning and visuospatial memory after MR stimulation [56]. In healthy individuals, no such age-depended differences were observed, and both younger and elderly participants performed better after MR stimulation compared to placebo [33]. With respect to the salience of depression-related emotional stimuli, there was no effect of MR stimulation on selective attention or facial emotion recognition, neither in MDD nor in healthy controls [57]. Interestingly, MDD with psychotic symptoms or treatment resistance showed more pronounced MR alterations [58, 59], suggesting that MDD subgroups differ in MR functioning and its association with cognition. Until now, there are too few studies to draw final conclusions regarding the association between cognition and MR functioning in MDD.

In contrast to MDD, meta-analyses of HPA-axis functioning in PTSD suggest reduced rather than enhanced basal cortisol concentrations [60, 61]. However, results are inconsistent across studies, and there are several potentially influencing factors, such as differences in trauma type, symptom patterns, sex, genetic factors, time of cortisol measurement and comorbidity with other mental disorders such as MDD [61, 62]. Additionally, enhanced HPA-axis feedback sensitivity and enhanced GR sensitivity have been reported [63, 64]. Others emphasize the evidence for enhanced central activity of hypothalamic CRF in PTSD, which is supported by a blunted ACTH response to exogenous CRF, possibly due to a down-regulation of pituitary CRF receptors [65]. In sum, a CRF overdrive in PTSD in concert with reduced cortisol release has been proposed. Furthermore, lower cortisol measured shortly after the occurrence of trauma was associated with the development of PTSD, suggesting that low cortisol concentrations might be a pre-existing risk factor [64]. Hypersensitivity of the GR might, in turn, be a consequence of reduced cortisol availability.

Assuming that PTSD patients exhibit GR hypersensitivity would lead to the expectation of stronger effects of acute stress and cortisol on cognition compared to healthy individuals. Indeed, one study reported a stronger negative effect of cortisol on declarative memory in PTSD compared to controls [66]. Interestingly, comorbid BPD (which affected 50% of PTSD patients in this sample) was associated with overall better memory performance, which equally decreased after hydrocortisone stimulation. Furthermore, PTSD patients showed impairments in working memory after GR stimulation compared to healthy controls [66]. Cortisol also impaired associative learning in PTSD patients but not in the control group [67]. A study from our group shows opposing effects of cortisol on memory when comparing PTSD patients with healthy individuals [68]. Cortisol impaired memory retrieval in the control group but enhanced declarative and autobiographical memory retrieval in the PTSD group. Put differently, the differences between PTSD patients and controls in memory performance, as observed in the placebo condition, diminished after cortisol administration. An enhanced working memory performance after the injection of cortisol was also observed in older patients with PTSD [69]. A neuroimaging study with veterans suffering from PTSD found enhanced hippocampus activation after administration of cortisol, which was not seen in control veterans without PTSD [70]. In sum, these results support the hypotheses of enhanced GR sensitivity in PTSD, with stronger effects of GCs on cognition. This pattern strongly differs from a lack of GC effects observed in patients with MDD. These differences have to be kept in mind when evaluating the findings of BPD.

### 2.4 The HPA-axis in BPD

BPD patients show a very high comorbidity with MDD (32% to 83%) and PTSD (25% to 56%, median of several cross-sectional studies is 46.9%) [2], two mental illnesses for which different alterations of the HPA-axis have been repeatedly described. Some studies have directly investigated HPA-axis alterations in BPD. Compared to other mental disorders, however, relatively few studies exist.

A recent meta-analysis that included single-measurement investigations of basal cortisol release did not find alterations in BPD patients [71]. In contrast, studies with multiple cortisol assessments, *e.g*., by measuring salivary [72, 73] or urinary cortisol several times during the day [74], suggest enhanced cortisol release in BPD, which also reached meta-analytic significance [71]. However, the comparability of studies is restricted due to the high heterogeneity of employed methods. Furthermore, dissociative symptoms, as well as comorbid PTSD and depressive symptoms, were repeatedly shown to influence results [74-77].

As the function of GC receptors is essential for the cognitive effects of cortisol, measurements of the related receptor status are of interest. But again, studies on BPD are scarce. One study measured the GR gene methylation status and suggested dysfunction of the GR [78, 79]. Our group measured the ability of corticosteroids to inhibit T-cell proliferation but did not find any differences between BPD patients and controls regarding MR and GR sensitivity [80].

Most studies that investigated HPA-axis feedback regulation in BPD used the standard 1 mg dexamethasone suppression test (DST). Dexamethasone is a synthetic cortisol derivative applied orally or intravenously to test if it activates the HPA-axis feedback mechanism by binding at GC receptors and initiating a suppression of ACTH release, subsequently leading to reduced cortisol levels. The majority of these older studies reported higher cortisol concentrations after dexamethasone or high rates of non-suppressors compared to control [81-85], suggesting reduced HPA-axis feedback sensitivity in BPD. As already mentioned, comorbid depressive systems strongly influence HPA-axis feedback regulation, and most of these early studies showed effects of affective symptoms or comorbid MDD. For instance, BPD patients with comorbid MDD had higher cortisol levels after dexamethasone administration compared to those without comorbid MDD [86], which could be attributed to the known effect of reduced HPA-axis feedback in MDD. When compared to patients with MDD, BPD patients showed less non-suppression to dexamethasone, which might speak for differences in HPA-axis functioning [87, 88]. However, not all of these earlier studies applied sufficient diagnostic procedures to assess BPD symptomatology, making the data difficult to interpret.

In addition to depressive symptoms, the role of comorbid PTSD in HPA-axis feedback regulation in BPD has been emphasized [86]. To detect the hyper-suppression to dexamethasone as indicative of a hyper-sensitive HPA-axis feedback mechanisms in PTSD, the use of a low-dose (0.5 mg) DST has been advocated [89]. Here first evidence suggests enhanced cortisol suppression in BPD [90, 91], but again, not all studies agree [72, 73]. However, evidence is emerging that trauma-related symptoms lead to differences in HPA-axis feedback regulation in BPD, although the observed effects are not consistent [92-94].

At this point, an inherent problem regarding the investigation of the HPA-axis function in BPD becomes apparent. While both MDD and PTSD comorbidities are frequent in BPD, both conditions are characterized by opposing HPA-axis alterations: while MDD seems to reduce HPA-axis sensitivity, PTSD is associated with increased feedback sensitivity. At first glance, excluding these comorbidities seems to offer a solution to this problem. However, such overly exclusive study populations would not be representative of the BPD population as a whole, thus running into another methodological problem.

Most studies mentioned above investigated well-defined basic cognitive processes within controlled laboratory settings. In reality, cognitive functioning occurs in the context of social interaction, which can be particularly stressful for BPD patients. As such, situational social demands can lead to additional acute social stress, which might further affect cognitive and social functioning. It is, therefore, important to consider how acute stress affects social cognition and functioning in BPD.

## 3 STRESS AND SOCIAL COGNITION

In animal models, high and acute stress is associated with the (freeze)-fight-or-flight response promoting aggression, withdrawal or dissociation. In humans, on the contrary, acute stress can lead to prosocial effects, in particular regarding social cognition and behaviour. The tend-and-befriend hypothesis suggests an (evolutionary) adaptive role in repairing social bonds after conflicts or increasing social cohesion in one’s group [95]. The concept of tend-and-befriend predicts enhanced prosocial behavior in response to a psychosocial stressor instead of the well-described fight-or-flight response [96].

Recent research supports the prediction that acute stress impacts social cognition in healthy participants. Deckers, and Lobbestael [97] reported an increase in emotion recognition performance after the Trier Social Stress Test (TSST), a well-validated psychosocial stress paradigm (see **Box 1**). Furthermore, MR stimulation increased attentional bias to negative facial expressions [98]. Additionally, self-reported emotional empathy increased after psychosocial stress as well as MR stimulation [99-101]. These results fit nicely with the hypothesis that acute (psychosocial) stress leads to increased prosocial tend-and-befriend behavior [102, 103]. In line with these assumptions, healthy young men showed enhanced trust, trustworthiness and sharing behavior after TSST, while no effect of stress on punishment behavior was observed [95]. However, other studies reported reduced punishment and less trust after TSST [104].

A recent review [105] finds no clear direction of stress effects on prosocial behavior, arguing that the context determines whether participants select altruistic or competitive strategies to achieve their goals. The authors explain the increase in cognitive empathy as a shift from executive control towards a salience network, which seems to increase attention to the emotions of others. However, an increase in social orientation and empathy after acute stress might also be a coping mechanism employed to reduce perceived threats by increasing social cohesion. Accordingly, the direction between stress and empathy is bidirectional: Not only does acute stress mobilize an increase in empathy, but connecting empathetically with another person can reduce one’s own stress and cortisol response [106].

A recent meta-analysis did not identify a consistent effect of stress and cortisol release on prosocial behaviour in economic games that involve trust and sharing [107]. This might suggest that the downstream influence of stress on social cognition is not solely driven by the HPA axis. The ANS is immediately activated upon stress, leading to the release of noradrenaline from the locus coeruleus projections in the brain and, in turn, the release of adrenaline in the periphery [17, 108]. The levels of catecholamines are known to increase rapidly and normalize not long after the stressor offset [108]. External activation of the HPA-axis also produced a reduced vagal tone, thus affecting the parasympathetic branch of the ANS [109]. This speaks for a dynamic interplay between HPA-axis and the ANS, with both systems interacting and potentially affecting different cognitive functions [110]. In line, a recent meta-analysis showed that emotional control, *i.e*., an executive function that requires cognitive control but not effortless and automatic emotion recognition and empathy, was related to cortisol levels [111]. As such, emotional and cognitive processes might rely on partly distinct, yet potentially interacting, neuro-physiological mechanisms.

The present section (3.1.) will summarize key aspects of social cognition and how they are affected by BPD. Then (3.2.) the effect of acute stress, and potentially the HPA-axis, on social cognition in BPD will be reviewed, focusing on studies using two well-established paradigms of stress-induction, the (3.2.1) Trier Social Stress Test and (3.2.2.) the Cyberball task.

### 3.1 Social Cognition in BPD

Healthy individuals might react to interpersonal stress by re-engaging with their social group in order to re-establish social cohesion. Their stress response thus promotes social cognition. In BPD patients, on the other hand, changes in the stress response, potentially stemming from HPA-axis dysregulation, might be associated with decreased social functioning. It has repeatedly been shown that social cognition, which is the process of adequately perceiving and processing social signals, is impaired in BPD [112].

A prerequisite for appropriate responses and functioning in interpersonal relationships is the ability to adequately process social signals, such as facial expressions of emotions. Findings regarding facial emotion recognition (FER) in BPD patients are heterogeneous and range from enhanced abilities [113, 114] to no differences compared to healthy controls [115, 116] to impaired FER capabilities (*e.g*., [117-119]). A large meta-analysis found FER deficits for intense expressions of anger and disgust and a bias in the perception of neutral stimuli among BPD patients [117]. This is in line with other studies, suggesting a hypersensitivity to angry faces [120-122], a negativity bias when judging positive facial expressions [123] and deficits in the discrimination of happiness as well as slow reaction times when processing happy faces [120, 122]. In sum, evidence suggests that BPD patients do not experience general deficits in FER but rather subtle impairments or hypersensitivity to potentially threatening stimuli, such as angry faces. Furthermore, they seem to be more confident in their evaluations, potentially suggesting a difficulty in representing or tolerating ambiguity and uncertainty [116].

Another closely related concept of social cognition that is relevant to BPD is rejection sensitivity (RS). It can be defined as a disposition to anxiously expect, readily perceive, and intensely react (emotionally or behaviorally) to signals of interpersonal rejection [124, 125]. There is broad evidence that individuals with BPD show pervasive and inflexible expectancies of rejection across several situations [126, 127]. Accordingly, BPD patients tend to interpret the reactions of others as hostile and negative [128-130].

Empathy is a further important component of social cognition and can be divided into cognitive and emotional empathy. Cognitive empathy refers to the ability to correctly infer or identify others’ mental states and is closely related to the concepts of the theory of mind or mentalizing [131]. BPD patients show profound deficits in the theory of mind, including difficulties identifying and reasoning about other people’s mental states [132]. They performed more slowly and made more errors on the false belief task, the performance of which crucially relies on intact self-other discrimination [133]. Emotional empathy, on the other hand, constitutes the own emotional response to another person’s emotional state. A recent meta-analysis reports deficits of empathy among BPD patients in eighty percent of all included studies [134]. On the other hand, studies using the Interpersonal Reactivity Index, a self-rated questionnaire of emotional empathy [135], suggest higher scores in BPD patients. However, it has been argued that this pattern stems from the increased emotional contagion of BPD patients, leading them to absorb the emotional state of others without the possibility to represent the emotion of others as independent and possibly even different from one’s own [136].

Inconsistent findings regarding social cognition might further be explained by comorbid psychiatric disorders. Especially childhood trauma or post-traumatic stress disorder (PTSD) has been shown to influence social cognition, such as empathy, in patients with BPD [112, 137]. Additionally, differences in methodology or contextual factors, such as the extent of (social) stress during the experimental situation, seem to play an important role.

### 3.2 Stress and Social Cognition in BPD

BPD patients are particularly prone to interpersonal stress and experience high subjective interpersonal difficulties in social situations, including experimental laboratory settings. It seems plausible that acute stress in general, and potentially HPA-axis dysregulation in particular, contributes to decreases in social cognition in BPD, as opposed to the increases in social cognition observed in healthy participants and suggested by the tend-and-befriend hypothesis.

Two experimental paradigms that have frequently been used to induce acute social stress in controlled laboratory environments, allowing reliable and repeated measures of physiological and psychological parameters, are The Trier Social Stress Test (TSST) and the Cyberball paradigm (see **Box 1**). Importantly, while both paradigms induce acute social stress, only the TSST has been shown to reliably increases cortisol release [138], while the Cyberball task did not increase cortisol levels in healthy participants [139-141]. This difference is useful to evaluate whether a potential effect of acute social stress on social cognition in BPD is driven by the HPA-axis or not.

Table **1** summarizes studies that used the TSST to investigate (1) social cognition, that is, FER, RS or empathy, and (2) HPA-axis response to stress as indicated by cortisol reactivity. As a juxtaposition, we also report the effects of the social stress induced by Cyberball on social cognition in BPD patients to disentangle the potential effects of HPA-axis dysregulations from mere social stress in the absence of an HPA-axis response.

#### 3.2.1 Studies using the TSST

Regarding HPA axis activation, most TSST studies find a blunted cortisol response in female BPD patients to social stress [97, 150-154] or no differences compared to healthy controls [101, 115, 155, 156] (see also Table **1** - column “HPA-axis stress reactivity”). One study suggested that higher trait dissociation might lead to higher cortisol-reactivity to TSST within the BPD population, although this analysis relies on a small subgroup sample [156]. One of the few studies that employed both male and female BPD patients further suggests potential sex-differences: while cortisol-levels were lowered in female patients compared to healthy controls, male BPD patients showed the opposite pattern [156]. However, overall, evidence regarding psychosocial stress in BPD supports the findings regarding stress in general discussed above, *i.e*., a blunted cortisol response [71].

In contrast to a blunted physiological stress response, almost all studies report increased perceived subjective stressfulness ratings in BPD [101, 154, 156], indicating that BPD patients experienced the TSST as more psychologically stressful compared to healthy controls. Similarly, BPD patients reported more negative emotional states after TSST [97, 150, 153] as well as higher perceived threat and less controllability [153] (for more detail, see Table **1** – column “subjective stressfulness and emotional state”).

So far, only three studies have employed the TSST to study the effects of acute stress on social cognition in BPD, and all of them only recruited female patients. Table **1** briefly summarizes these results (right column “social cognition”).

Comparing the TSST with the P-TSST as a control condition, Graumann *et al.* [115] recently investigated FER in BPD patients. Patients did not differ from healthy controls regarding their abilities to identify emotions of sadness, anger or neutral faces. Furthermore, FER was not affected by acute social stress, thus showing no difference between TSST and P-TSST. Interestingly, in this sample, the BPD group also did not show differences in stress-induced reactivity of cortisol and salivary alpha-amylase, measures of HPA and ANS activity, respectively. Finally, BPD patients showed overall more negative emotions compared to healthy controls. However, these were not affected by social stress.

Also, using the TSST, Deckers and Lobbestael [97] showed that the BPD group did not differ from a cluster C personality disorder sample (including patients showing avoidant, dependent and obsessive-compulsive traits) or healthy participants in how they evaluated the experimenter’s character, another aspect of social evaluation and cognition. Although BPD patients rated the experimenter as more hostile and less reliable even before the stressor, stress did not produce any further group differences. As such, after the TSST, all groups equally ascribed more negative traits to the experimenter than before TSST. Similarly, BPD patients did not differ from the clinical and non-clinical control group regarding their performance in a FER task which required the identification of anger, disgust, sadness, surprise and happiness. All groups performed better after TSST than before. However, since the study lacked a control condition (such as P-TSST), this difference might also be attributed to learning effects.

Wingenfeld *et al.* [101] used the Multifaceted Empathy Test (MET) to investigate the effect of TSST on cognitive and emotional empathy. While social stress did not affect cognitive empathy neither in BPD patients nor in healthy controls, the TSST led to increased emotional empathy in healthy controls but not in BPD patients. This is an interesting contrast to the finding that MR stimulation increased emotional empathy in BPD patients and healthy controls alike [100]. The present results thus suggest a stress-related increase in social cognition in healthy controls in line with the tend-and-befriend hypothesis. In contrast, social cognition in BPD patients was not affected by interpersonal stress. However, social stress led to stronger perceived stressfulness in BPD patients compared to healthy controls. Interestingly, these effects did not depend on the stress-related cortisol response, which did not differ between groups.

Taken together, the reviewed TSST studies do not support the claim that BPD patients are particularly prone to stress-induced dysregulation in social cognition. Neither the ability to recognize others’ emotions nor the extent to which these emotions evoked an empathetic response was affected by the TSST. It thus seems unlikely that the blunted HPA response induced by social stress in BPD significantly affected social cognition in BPD patients. However, neither did social stress facilitate social cognition in BPD patients, as it did in healthy participants [101] and after MR stimulation [100]. As such, even though BPD patients did not explicitly show signs of fight-or-flight behavior, social stress did not activate tend-and-befriend behavior in BPD patients either, even though subjectively, BPD patients perceived the social situations as particularly stressful compared to healthy controls.

#### 3.2.2 Studies using the Cyberball Task

As mentioned above, rejection sensitivity (RS) is an important aspect of social cognition, as it can lead to systematically and persistently biased perceptions of social situations. The Cyberball task creates a social situation in which different aspects of RS can be investigated. While the exclusion condition can induce acute feelings of rejection, the inclusion condition creates an objectively fair social interaction that can reveal persistently biased social cognition in terms of RS.

Staebler *et al.* [157] showed that BPD patients felt more excluded during Cyberball, regardless of condition, that is, even when they were included. After exclusion but not inclusion, BPD patients reported stronger other-focused negative emotions, a pattern that was absent in healthy participants. This might suggest a perceptual shift towards the external environment in line with the fight-or-flight hypothesis. Furthermore, compared to inclusion, during exclusion, BPD patients were expressing more mixed emotions (*e.g*., covering a negative emotion by smiling).

Renneberg *et al.* [158] demonstrated a biased perception of the amount of social inclusion in BPD: patients reported that they received fewer ball tosses and felt more excluded than healthy controls. However, no effect of the Cyberball condition emerged, again showing that this general RS effect was not affected by the objective level of inclusion. Both groups showed an increase in anger after the exclusion, while BPD patients also showed a decrease in sadness after both Cyberball conditions, which might suggest a shift from self-focused sadness to externally oriented anger in the context of social stress (see also [159]).

Interestingly, in a new Cyberball paradigm using partial exclusion by only one of the two co-players, BPD patients showed a trend towards punishing the excluder, which is in line with a fight-and-flight response. Healthy controls, on the other hand, tended to pass the ball slightly more often to the excluder, which is in line with a tendency to tend-and-befriend [160].

Overall, studies employing Cyberball quite consistently show stronger feelings of ostracism in BPD patients compared to healthy controls, both after exclusion [159, 161, 162], but, importantly, even after inclusion [148, 163-165]. Only when an overinclusion condition was used BPD patients’ emotional responses align with those of healthy controls, yet even then, BPD patients showed lower feelings of connection [147] and higher need threat [148, 166].

While Cyberball has been repeatedly used to study RS, only one recent study from our group employed the Cyberball task to study empathy in BPD patients [166]. In a large sample of female patients with BPD and tightly matched control participants, the MET was employed to compare cognitive and emotional empathy after either the exclusion of overinclusion condition of the Cyberball. Cognitive empathy, which shows some conceptual overlap with FER, did not differ between groups or Cyberball conditions. Compared to healthy controls, women with BPD reported lower emotional empathy for positive but not negative emotions. Exploratory analyses suggested that this effect might be more pronounced after social exclusion compared to overinclusion, but this effect of social stress was relatively small. While this might tentatively indicate decreasing emotional empathy in BPD following acute social stress, the overall results suggest that psychosocial stress is not the critical aspect that reduces emotional empathy in women with BPD. More research is needed to replicate these findings and further investigate how Cyberball affects other measures of social cognition in BPD patients.

Regarding HPA-axis activation during Cyberball, Jobst [159, 167] and Graumann [166] showed that the exclusion condition of the Cyberball did not affect cortisol levels, and, importantly, BPD patients did not differ from healthy controls. As such, the effects of Cyberball on social cognition are not likely to stem from a potential dysregulation of the HPA-axis in BPD.

## CONCLUSION

We reviewed how social stress induced by TSST and Cyberball affects social cognition in BPD patients as compared to healthy controls and to what extent this relationship is affected by the HPA axis. The first finding is that TSST, comparable to other (non-social) stressors, mainly revealed a blunted cortisol response in BPD patients compared to healthy participants. Interestingly, measurements of markers of the autonomic nervous system also hint towards a blunted reactivity to acute stress in BPD [150, 151, 153, 154], but not all studies concur [152, 155, 156]. Cyberball, as expected, did not show a HPA-axis response, neither in BPD patients nor in healthy controls. In contrast to the blunted response of the HPA-axis, BPD patients’ subjective experience of stress was consistently higher, and so was the extent of the accompanying negative affect.

More surprisingly, the reviewed literature does not reveal a clear effect of acute social stress on social cognition in BPD patients. While studies using the Cyberball task showed a persistent bias towards feeling socially excluded, studies using the TSST showed that social stress did not lead to changes in emotion recognition or empathy in BPD. However, evidence is still scarce, as only three TSST studies explicitly targeted social cognition, of which two measured FER and one empathy, thereby only covering a limited range of socio-cognitive functions. More research with sufficiently powered designs is needed to confirm and extend these findings, preferably including a wider range of measures of social cognition, for example, targeting mentalizing and theory-of-mind functioning, but also measures of prosocial behavior.

The lack of a consistent relationship between acute social stress on social cognition makes it difficult to evaluate whether the HPA axis plays a role in the relationship between stress and social cognition in BPD. However, it leaves the possibility that sustained HPA-axis dysregulation might contribute to difficulties in social situations even in the absence of acute social stress. It also seems plausible that not only the HPA axis function affects social cognition in BPD, but social functioning rather relies on a balance between several psychophysiological mechanisms. As already mentioned, the ANS plays a relevant role in promoting fight-and-flight *vs.* tend-and-befriend behavior. Besides the ANS, recent evidence suggests that the endocannabinoid system is involved in the homeostatic regulation of the HPA-axis [168], with first studies demonstrating generally lowered endocannabinoid levels in BPD [169], which directly correlate with cortisol levels [170]. Furthermore, the oxytocin system plays an important role in regulating feelings of social attachment and trust, which seem very relevant for social interaction [171]. In fact, first evidence suggests a differential oxytocin response to social exclusion during the Cyberball task in BPD compared to HC [159]. Future research is needed to clarify the differential mechanisms (and their respective interactions) involved in the response to acute (social) stress in BPD.

The observations above contrast the phenomenological experience of BPD patients, who often report enhanced subjective distress in social situations. As such, maybe one of the most consistent stress-induced effects in BPD does not relate to social cognition but is the subjective increase in negative mood and distress both after TSST and Cyberball. Thus, irrespective of acute social stress, the question of whether a non-sufficient (blunted) physiological stress response is associated with a heightened perception of distress needs further research. One hypothesis might be that the lower physiological stress-response of BPD patients prevents the activation of adaptive tend-and-befriend behavior, which could instead promote social cohesion and reduce stress as observed in healthy controls. A deficient activation of the endocannabinoid and/or oxytocin system to (down-) regulate HPA activation during acute stress might also be a promising target for future research.

All in all, more research is needed to better understand the acute stress response of patients with BPD. Furthermore, several limitations of the existent findings have to be acknowledged. Most of the reviewed studies only included female BPD patients. This prevents the generalizability of the findings to the male population. In fact, the few studies using a mixed population hint at existing sex differences. In females, further interactions of the stress response with fluctuating sex hormones are possible, and only a few studies restrict testing to the luteal phase. Finally, as stated above, high comorbidity with MDD and PTSD makes it difficult to study the specific stress signature of BPD in isolation.

## Figures and Tables

**Box (1):**
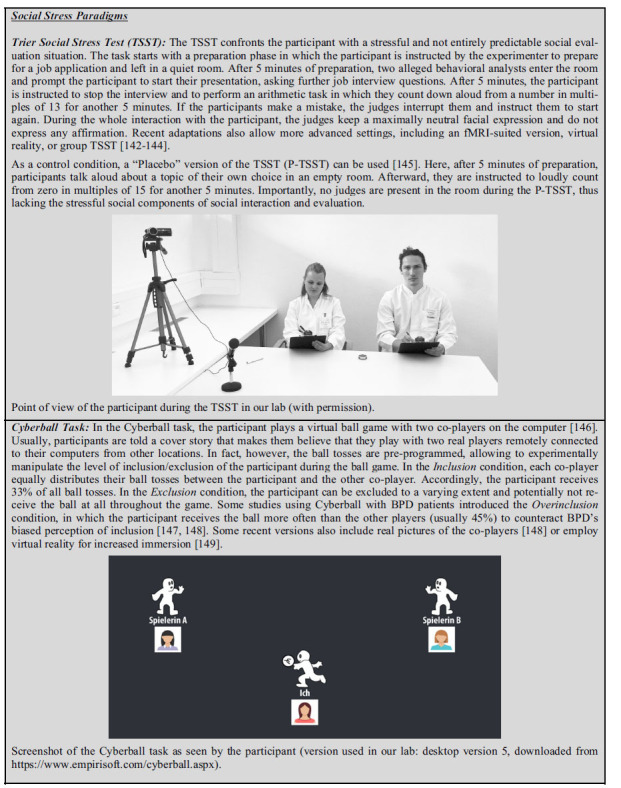
Social stress paradigms.

**Table 1:** Overview of studies using the TSST in BPD to investigate social cognition and/or cortisol reactivity.

**Study**	**Sample *n***	**Psychotropic Medication**	**Comorbidities (MDD/PTSD)**	**Induction of Stress**	**Dependent Measures**		
					**HPA-axis Stress Reactivity**	**Subjective Stressfulness and Emotional State**	**Social Cognition**
Aleknaviciute *et al*. (2018) [150]	**BPD** = 26	0%	MDD = 0% PTSD = 30%	- Stress: TSST - Control: none	**Cortisol Reactivity:** - Stress-induced increase across groups. - Blunted response in BPD *vs.* CPD and HC.	**Emotional State (POMS):** - More negative mood after stress across groups. - Increase in negative mood after stress higher for BPD/CPD *vs.* HC.	-
	**CPD** = 20	0%	MDD = 0% PTSD = 5%				
	**HC** = 35	-	-				
Deckers *et al*. (2014) [97]	**BPD** = 22	45%	MDD = 32% PTSD = 23%	- Stress: TSST - Control: none	**Cortisol Reactivity:** - Blunted response in BPD and CPD *vs.* HC.	**Emotional State (POMS):** - More negative mood after stress across groups. - Increase in negative mood after stress higher for BPD *vs.* HC.	**Social evaluation of experimenter (borderline symptom-related, negative, neutral cognitions):** - After stress increase of all types of cognitions across groups. **FER (anger, disgust, fear, happiness, sadness, surprise):** - Improved after TSST across groups.
	**CPD** (cluster C Personality disorder) = 23	48%	MDD = 27% PTSD = 9%				
	**HC** = 24	-	-				
Duesenberg *et al*. (2019) [151]	**BPD** = 49	67%	MDD = 0% PTSD = 45%	- Stress: TSST - Control: P-TSST (cross-over design)	**Cortisol Reactivity:** - Increase after TSST but not after P-TSST across groups. - Increase from before to after TSST slightly blunted in BPD *vs.* HC.	**Mood States (MDMQ):** - More negative mood after TSST *vs.* P-TSST across groups. - More negative mood after TSST in BPD *vs.* HC.	-
	**HC** = 49	-	-				
Ehrenthal *et al*. (2018) [155]	**BPD **= 39	*Data available on request*	*No information*	- Stress: TSST - Control: none	**Cortisol Reactivity:** **-** Stress-induced increase across groups. - No group effect (BPD = HC).	-	-
	**Some BPD criteria **= 15						
	**HC **= 59						
Graumann *et al*. (2021) [115]	**BPD** = 43	70%	MDD = 0% PTSD = 42%	- Stress: TSST - Control: P-TSST	**Cortisol Reactivity:** - Higher increase after TSST *vs.* P-TSST. - No group effect (BPD = HC).	**Mood States (MDMQ):** - More negative mood after TSST *vs.* P-TSST. - The more negative mood in BPD *vs.* HC.	**FER (Sadness, Anger, Neutral):** - No stress effect (TSST = P-TSST). - No group effect (BPD = HC).
	**HC** = 46	-	-				
Inoue *et al*. (2015) [152]	**BPD** = 72	0%	MDD = 0% PTSD = 0%	- Stress 1: TSST - Stress 2: electric stimulation test (cross-over design)	**Cortisol Reactivity:** - Blunted response after TSST in female BPD *vs.* female HC. - Increase after TSST in male BPD *vs.* male HC.	**Emotional State (POMS):** - More negative mood before stress in BPD *vs.* HC.	-
	**HC** = 377	-	-				
Nater *et al*. (2010) [153]	**BPD** = 15	0%	MDD = 0% PTSD = 33%	- Stress: TSST - Control: none	**Cortisol Reactivity:** - Blunted response in BPD *vs.* HC. **Adrenocorticotropic ** **Hormone Reactivity:** - Stress-induced increase across groups. - ACTH-to-cortisol ratio higher in BPD *vs.* HC.	**Primary Appraisal Secondary Appraisal (PASA):** - Higher perceived threat and lower controllability of stress in BPD *vs.* HC. **Subjective Stress Rating:** - No group effect after stress (BPD = HC).	-
	**HC** = 17	-	-				
Scott *et al*. (2013) [154]	**BPD** = 33	79%	Mood disorders: 21%	- Stress: TSST - Control: none	**Cortisol Reactivity:** - Blunted response in BPD *vs.* HC-m/HC-n. - Equal decrease during recovery across groups.	**Negative Affect (PANAS-NA):** - Equal elevation and recovery after stress across groups. **Subjective Stress Perception Rating Form (SSPRS):** - Higher after-stress in BPD/HC-m *vs.* HC-n.	-
	**HC-m** = 27 matched for negative affect and impulsivity.	-	-				
	**HC-n** = 30 non-matched	-	-				
Simeon *et al*. (2007) [156]	**BPD-hi** = 5 with high trait dissociation	0%	MDD = 0% PTSD = 0%	- Stress: TSST - Control: none	**Cortisol Reactivity:** - Higher increase in BPD-hi *vs.* BPD-lo/HC. - No group effect (combined BPD = HC). - No sex effect. **Norepinephrine Reactivity:** - No group effect (combined BPD = HC).	**Emotional State (POMS):** - Negative mood in BPD-hi > BPD-lo > HC before and after stress (contrasts not reported). **Subjective Stress Rating:** - Slightly higher after stress for combined BPD *vs.* HC.	-
	**BPD-lo** = 8 with low trait dissociation	0%	MDD = 0% PTSD = 0%				
	**HC** = 11	-	-				
Wingenfeld *et al*. (2018) [101]	**BPD** = 47	70%	MDD = 0% PTSD = 43%	- Stress: TSST - Control: P-TSST	**Cortisol Reactivity:** - Higher increase after TSST *vs.* P-TSST. - No group effect (BPD = HC).	**Subjective Stress Rating:** - Higher after TSST *vs.* P-TSST. - Higher after TSST in BPD *vs.* HC.	**Multifaceted Empathy Test (MET)** Cognitive empathy: - No stress effect (TSST = P-TSST). - No group effect (BPD = HC). Emotional empathy: - Lower in BPD *vs.* HC after TSST but not after P-TSST.
	**HC** = 47	-	-				

**Abbreviations:** BPD = borderline personality disorder; HC = healthy controls; CPD = cluster C personality disorder; MDD = major depressive disorder; PTSD = posttraumatic-stress disorder; HPA-axis = hypothalamic-pituitary axis; TSST = Trier Social Stress Test; P-TSST = Placebo Trier Social Stress Test; POMS = Profile of Mood States; MDMQ = Multidimensional Mood State Questionnaire; PASA = Primary Appraisal Secondary Appraisal; SSPRS = Subjective Stress Perception Rating Form; PANAS-NA = Positive and Negative Affect Schedule – Negative Affect; FER = facial emotion recognition; MET = Multifaceted Empathy Test.

## LIST OF ABBREVIATIONS

ACTH: Adrenocorticotropin
ANS: Autonomic Nervous System
BPD: Borderline Personality Disorder
CRF: Corticotropin-releasing Factor
DBT: Dialectical Behavior Therapy
GCs: Glucocorticoids
GR: Glucocorticoid Receptor
HPA: Hypothalamic-pituitary-adrenal
MBT: Mentalization-based Therapy
MDD: Major Depressive Disorder
MR: Mineralocorticoid Receptor
PFC: Prefrontal Cortex
PTSD: Posttraumatic Stress Disorder
RS: Rejection Sensitivity
TSST: Trier Social Stress Task
